# Molecular Characterization of a Beak and Feather Disease Virus Genome from a Purple Crowned Lorikeet (*Glossopsitta porphyrocephala*)

**DOI:** 10.1128/genomeA.01102-16

**Published:** 2016-10-06

**Authors:** Shubhagata Das, Sarker Subir, Kathleen Fearnside, Jade K. Forwood, Seyed A. Ghorashi, Shane R. Raidal

**Affiliations:** a School of Animal and Veterinary Sciences, Charles Sturt University, Wagga Wagga, New South Wales, Australia; b Department of Physiology, Anatomy and Microbiology, School of Life Sciences, La Trobe University, Melbourne, Victoria, Australia; c Normanhurst Veterinary Practice, Normanhurst, New South Wales, Australia; d School of Biomedical Sciences, Charles Sturt University, Wagga Wagga, New South Wales, Australia

## Abstract

The complete genome sequence of beak and feather disease virus (BFDV) from a purple crowned lorikeet (*Glossopsitta porphyrocephala*) was characterized. The genome consists of 2,010 nucleotides and encodes replicase-associated protein and capsid protein. This is the first evidence of BFDV infectivity and complete genome sequence for this novel host.

## GENOME ANNOUNCEMENT

Psittacine beak and feather disease (PBFD) is a very common, chronic, and ultimately fatal viral disease of wild psittacine birds throughout the world, with all parrots, lorikeets, and cockatoos in endemic areas considered susceptible (1, 2). The causative agent of the disease, beak and feather disease virus (BFDV), belongs to the family *Circoviridae* (3) and is probably the dominant pathogen of wild *Psittaciformes*, with strong historical evidence of PBFD occurring naturally in wild Australian birds for more than 120 years (4, 5). BFDV itself is a nonenveloped icosahedral virus with an approximately 2.0-kb circular single-stranded DNA (ssDNA) genome that typically encodes two major proteins known as replication-associated protein (Rep) and capsid protein (Cap), with a potential stem-loop structure located between them (3, 6, 7). Although lorikeets are the members of a relatively young (10 Ma) parrot subfamily *Loriinae*, they represent one of the most robust or deeply host-adapted hosts of BFDV (8) and anecdotally have an inherent resistance to PBFD. Recent research reveals that despite much intralineage variation, the BFDV genomes circulating in lorikeets are genetically segregated and ancient compared to other contemporary BFDV lineages (9). In this study, we characterized a BFDV genome from a purple crowned lorikeet (*Glossopsitta porphyrocephala*) for the first time.

Postmortem collection of tissue samples was conducted from a captive purple crowned lorikeet (identification [ID], CS15-0956; year of sampling, 2015; location; Sydney, NSW, Australia) suspected to have *Psittacine adenovirus 1* infection. Histopathology and immunohistochemistry (IHC) was performed, which excluded adenovirus infection but revealed BFDV-positive antigen in spleen and bursa of Fabricius. Genomic DNA was extracted from the tissue samples according to established protocols (10, 11), and the whole-genome sequence was amplified using the primers and PCR conditions developed in previous studies (7, 9, 12).

The newly amplified BFDV genome (GenBank accession no. KX449320) comprises 2,010 nucleotides (nt), with a G+C content of 53.73%. The genome structure includes two major open reading frames (ORFs), ORF1 (nt 154 to 1026) and ORF2 (nt 1235 to 1984), encoding genes for Rep and Cap, respectively. Preliminary BLASTn (13) analysis of the assembled genome revealed 99% pairwise nucleotide match with one of the BFDV isolate from a rainbow lorikeet (*Trichoglossus haematodus*) (GenBank accession no. KM887929) (9). Separate BLASTn searches for the *rep* and *cap* genes also demonstrated a similar result. However, subsequent BLASTn search of the *cap* gene showed 99% pairwise match with a different isolate (GenBank accession no. KM887937), which represents recombination among the isolates. The overall nucleotide identity of the new BFDV isolate ranges from 88 to 99% compared to the BFDV genomes available on GenBank. However, an initial phylogenetic analysis demonstrated that this newly assembled genome is positioned in the lorikeet-specific clade of BFDV genealogic tree with strong consensus support. This is the first report of a BFDV genome identification and characterization for this novel host species (*Glossopsitta porphyrocephala*), which may facilitate further research on viral evolution and recombination events in this host.

### Accession number(s).

The complete genome sequence of BFDV has been deposited at GenBank under the accession no. KX449320.
